# Stand-alone anterior cervical decompression and fusion surgery: A cohort study evaluating a shaped cage without plates or screws

**DOI:** 10.3389/fsurg.2022.934018

**Published:** 2022-09-21

**Authors:** Xiaolong Chen, Alisha Sial, Charmian Stewart, Jose Vargas Castillo, Ashish D. Diwan

**Affiliations:** ^1^ Spine Labs, St. George / Sutherland Clinical School, University of New South Wales, Kogarah, NSW, Australia; ^2^ Spine Service, Department of Orthopaedic Surgery, St. George Hospital Campus, Kogarah, NSW, Australia

**Keywords:** cervical spondylotic conditions, anterior decompression, fusion, stand-alone cage, complication

## Abstract

**Background:**

The anterior approach to the cervical spine is the most commonly used surgery with effective decompression and less surgical trauma. Anterior plate construct (APC) is considered a standard technique. However, it appears to cause implant failure and postoperative dysphagia. Due to these reasons, locking stand-alone cages (LSCs) without the addition of an anterior plate have been developed and gained popularity in the past decade. In theory, an LSC could provide immediate load-bearing support to the anterior column of the cervical spine and may enhance the rate of arthrodesis. However, screw skiving and backing off are known complications of LSC. Given the characteristic shape of cervical discs, we wondered whether there may be a role for a shape-conforming cage without screws and plates to achieve desired outcomes, i.e., a true stand-alone cage (TSC). A single surgeon cohort using the cage in a heterogenic set of indications was evaluated.

**Methods:**

A total of 45 patients with degenerative cervical conditions who underwent surgery using TSC using CoRoent Small Contoured peek cage (Nuvasive, San Diego, CA) and Orthoblend™ (Medtronics, Memphis, TN) were retrospectively reviewed. Comparisons between preoperative and postoperative Numeric Rating Scale (NRS), the modified AAOS-Modems disability outcome, Neck Disability Index (NDI) scores, and Short Form 36 were evaluated. Operative time, the occurrence rate of fusion, lordosis change of cervical spine, and occurrence rate of complications were evaluated.

**Results:**

There were one-level (*n* = 15), two-level (*n* = 24), and three-level (*n* = 6) cases making a total of 81 cages implanted and studied. The mean operative time was 132.7 min. The group demonstrated significant improvements in NRS, AAOS-Modems disability outcome, and NDI scores after surgery (mean follow-up 12 months). The cervical lordosis at pre- and last follow-up period was 8.7 ± 2.2° and 8.3 ± 3.2°, respectively. The complication rate was 21.2%.

**Conclusions:**

TSC yielded satisfactory long-term clinical and radiological outcomes; this preliminary report can form the basis of a cost–benefit analysis study either prospectively or by way of meta-modeling comparing APC, LSC to TSC.

## Introduction

Anterior cervical discectomy and fusion (ACDF) has been considered the standard surgical intervention for the treatment of cervical spondylotic conditions (e.g., a degenerative cervical disease with myelopathy or radiculopathy) (1, 2). The goal of this surgery is intended to obtain effective neural (e.g., spinal cord and nerve root) decompression, maintain the affected segment stabilization, and restore lordosis of the cervical spine (3, 4).

Anterior plate construct (APC) is a commonly used technique for ACDF (5). Traditionally, the anterior plate is used for maintaining the stabilization of the cervical spine, improving cervical lordotic alignment, increasing fusion rate, and preventing cage dislocation (6). However, the use of an anterior plate may lead to some potential adverse events, such as sore throat, dysphagia, implant failure, and adjacent segment degeneration (ASD). Due to these reasons, locking stand-alone cages (LSCs) without the anterior plate has been developed and gained popularity in the past decade (1). In theory, an LSC could provide immediate load-bearing support to the anterior column of the cervical spine and may enhance the rate of arthrodesis. Previous studies reported that LSC provided comparable stability and reduced the damage to soft tissues and plate-related complications with a satisfactory clinical outcome (7–12). Nevertheless, previously published studies showed that there were no advantages of LSC in clinical and/or radiologic outcomes and/or complications compared with APC (13, 14). Some complications following LSC have been reported, including screw skiving and backing off. Therefore, a consensus has not yet been arrived at on the efficacy of LSC in the reduction of neck pain and overall complications in cervical spondylotic conditions. Given the characteristic shape of cervical discs, we wondered whether there may be a role for a shape-conforming cage without screws and plates to achieve desired outcomes, i.e., a true stand-alone cage (TSC).

To further clarify arguments in the current literature, a single surgeon cohort using the cage (e.g., TSC) through a minimally invasive approach for treating the patients with a heterogenic set of indications was evaluated.

## Participants and methods

Ethical approval was obtained from the Human Research Ethics Committee of the University of New South Wales (NRR-HC210096) for the retrospective analysis of outcomes (e.g., demographic data, clinical outcome, and radiological outcome) of patients who have undergone stand-alone anterior cervical decompression and fusion surgery (TSC without the addition of an anterior plate) at Spine Service, St George Hospital Campus (UNSW Sydney, Australia).

### Design and patients

Inclusion criteria were the following: (1) age more than 18 years; (2) signs and symptoms of cervical spondylotic conditions (e.g., cervical radiculopathy or cervical spondylotic myelopathy); (3) cervical spondylotic conditions confirmed using magnetic resonance imaging; (4) patients signed the informed consent; and (5) at least of 3 months follow-up after surgery. Exclusion criteria were the following: (1) developmental cervical spinal stenosis; (2) ossification of the posterior longitudinal ligament; (3) systemic or local infection; (4) trauma, fracture, tumor, and invasive malignancy; and (5) surgical history of the cervical spine.

### Neurological assessment and clinical outcomes

Primary symptoms (e.g., symptoms for myelopathy or radiculopathy) including any hand–neck pain, clumsiness, radicular pain to the upper limb(s), leg stiffness, and gait disturbance were recorded. The Neck Disability Index (NDI) and Numeric Rating Scale (NRS) were used to assess disability and neck and radicular pain, respectively. The neck pain relief was rated with 6 points [score 1 = complete relief (100%); score 2 = small amount of symptoms persists (80%–99%); score 3 = most of symptoms are gone (60%–0%); score 4 = moderate relief (30%–60%); score 5 = minor relief (up to 30%); score 6 = no relief or symptoms worse]. Eighteen items were included in the modified AAOS-Modems disability outcome tool spine-service version for the physical functioning scale (PFS). Each item of this tool was manually rated with 5 points for one of three possible responses (score 0 = not limited at all, score 3 = little limitation, and score 5 = limited quite a lot). We obtained scores for the eight Short Form 36 (SF-36) subscales [physical functioning (PF), energy fatigue (EF), emotional wellbeing (EW), social functioning (SF), bodily pain (BP), general health (GH)]. All the data were collected preoperatively, at 1-month, 3-month, 6-month, 12-month, and last follow-up after surgery. The senior spinal surgeon with 30 years of experience (ADD) performed the neurological assessment and surgery.

### Surgical technique

**Figure 1:**
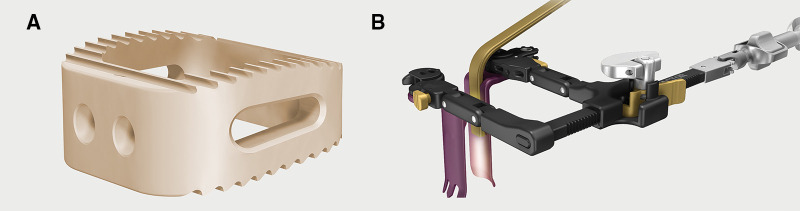
(**A**) CoRoent small contoured peek cage (Nuvasive, San Diego, CA). (**B**) Anterior retractors systems (Maxcess C retractor, Nuvasive San Diego CA).

Patients were placed in the supine position. The surgical procedure was exposed through a standard anterior approach from the left side. Small access corridors were used to minimize the damage to soft tissue. In order to obtain better visualization and illumination, the better anterior retractor systems (Maxcess C retractor, Nuvasive, San Diego, CA, Figure 1) were combined with the use of the loupes. This retractor system optimizes direct illumination using a cold light source directly attached to the retraction blade. Furthermore, the retractor is stabilized to the operating table diminishing needless retractor movement on soft tissue during the operation. For multilevel procedures, the retractors are moved one level at a time with segmental Casper pin distraction. Anterior cervical discectomy was performed. After dural and root decompression, patients underwent TSC using CoRoent Small Contoured peek cage (Nuvasive, San Diego, CA, Figure 1) and Orthoblend™ (Medtronics, Memphis, TN). The cages were filled with demineralized bone matrix for augmenting fusion.

The technique allows minimal dissection and smaller incisions, and allows for maximal spinal canal decompression and disc clearance through a minimally invasive technique.

### Radiological evaluation

**Figure 2:**
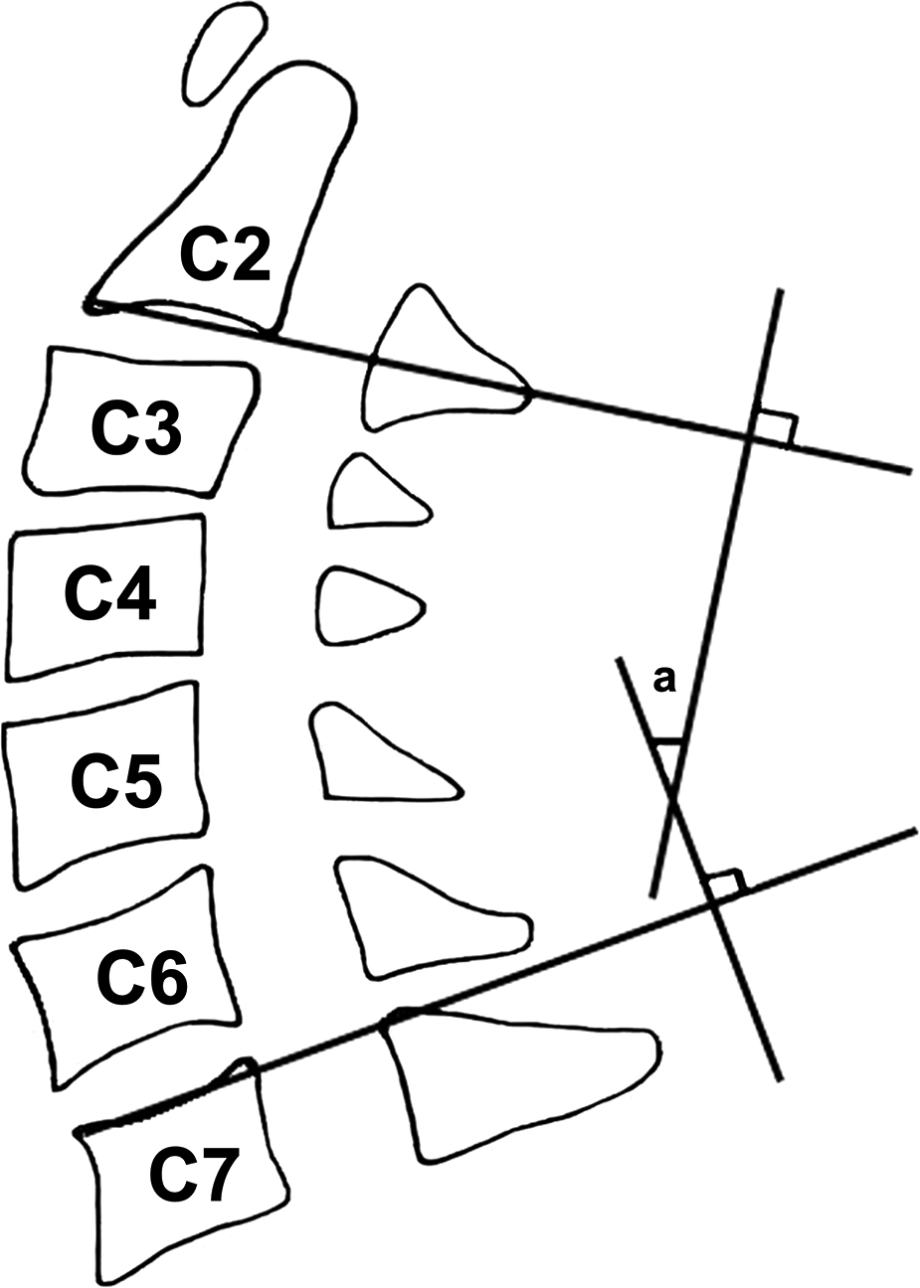
Cobb angle for measuring cervical lordosis. Cobb angle is measured on lateral x-ray of the lumbar spine: the angle (a) is formed by the inferior endplate of the C2 to the inferior endplate of the C7.

The preoperative and postoperative lordosis of the cervical spine, postoperative fusion rate, and postoperative subsidence were measured and evaluated *via* radiological images. The lordosis of the cervical spine was measured by the Cobb angle between the inferior endplate of C2 to the inferior endplate of C7 (Figure 2) (15). The definition of fusion was listed as (1) the range of motion of surgical level <2° in postoperative radiographs, (2) the formation of bridging trabecular bone between the involved vertebral bodies; and (3) the absence of a radiolucent gap through the fusion level. The incidence of subsidence was referred to as more than 3 mm reduction of the disc height in the involved level in postoperative images (16).

### Surgical complications

Procedure-related and postoperative complications at each follow-up time point were evaluated and collected by a clinical fellow (AS). Procedure-related complications include injury to recurrent laryngeal nerve, dural tear, nerve root damage, damage to the spinal cord, major blood vessel injury, infection, and damage to the trachea or esophagus. Postoperative complications include inadequate symptom relief after the surgery, pseudarthrosis, dysphasia, potential speech disturbance, hematoma, and ASD.

### Statistical analysis

The continuous variables were expressed as mean ± standard deviation (SD). Paired *t*-test was used to compare the clinical outcomes of NRS and NDI between preoperative and final follow-up. Due to the non-normal distribution of these data, the nonparametric Mann–Whitney *U* test was used to compare the NRS, NDI, PFS, PF, EF, EW, SF, BP, and GH between the preoperative and final follow-up groups. Categorical variable data were analyzed by Fisher's exact test. SPSS v24.0 (SPSS Inc., Chicago, IL, United States) was used for the statistical analysis. *P* < 0.05 was considered to be statistically significant.

## Results

### Patients

**Figure 3:**
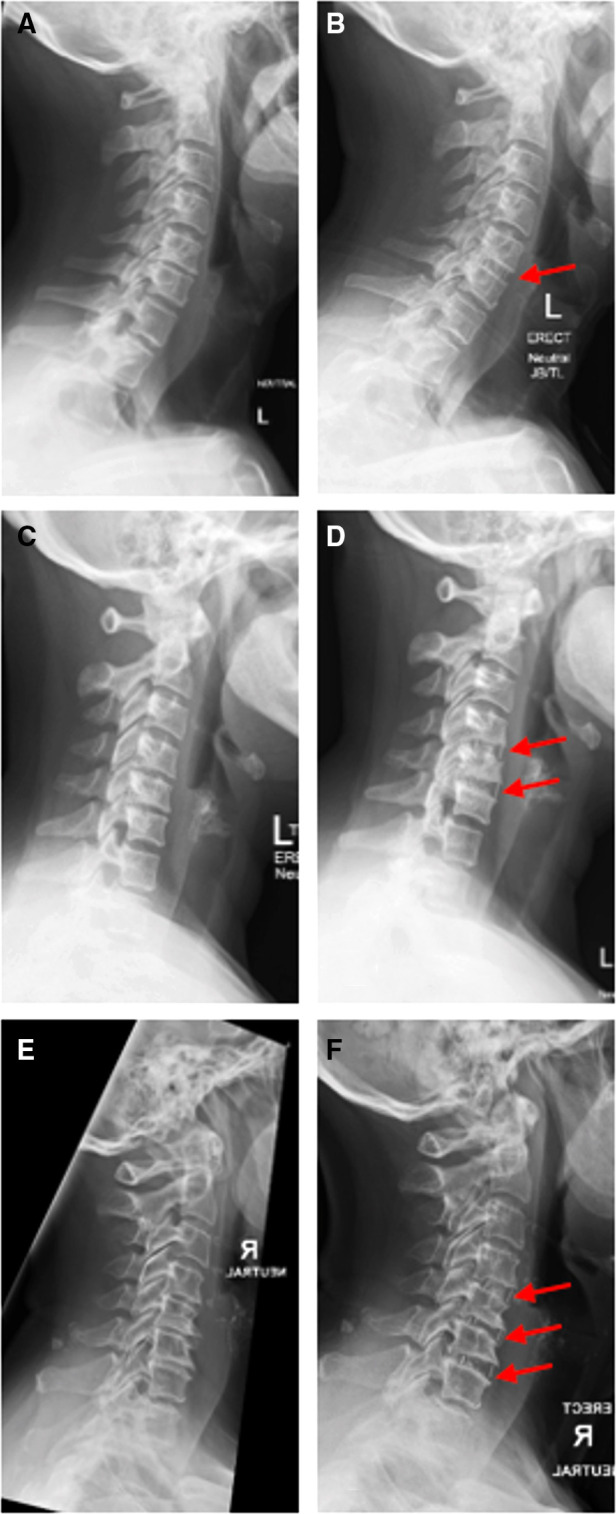
(**A,B**) Standing lateral x-ray of the true stand-alone cage for cervical degenerative disc disease in one-level (C5/6) preoperatively and at 2-year follow-up. (**C,D**) Standing lateral x-ray of the true stand-alone cage for cervical degenerative disc disease in two levels (C5/6 and C6/7) preoperatively and at 1-year follow-up. (**E,F**) Standing lateral x-ray of the true stand-alone cage for cervical degenerative disc disease of three levels (C4/5, C5/6, and C6/7) preoperatively and at 1-year follow-up.

This study included 45 patients (20 females and 25 males), aged 40–75 years (the mean age at surgery was 52.4 years), operated in our department by a senior surgeon (ADD) between November 2012 and January 2021, and having complete pre- and postoperative clinical and radiological data. The mean time to follow-up was 12 months (range 6–24 months). Fifteen cases with one-level, 24 cases with two-level, and six cases with three-level made a total of 81 cages implanted and studied (Table 1 and Figure 3).

**Table 1:** Demographics and clinical data.

Characteristic	Value
No. of patients	45
Mean of age (years)	52.4 ± 10.6
Female:male	20 (44.4%):25 (55.6%)
Indications	
No. of neck pain	45 (100%)
No. of radiculopathy	38 (84.4%)
No. of myelopathy	40 (88.9%)
Levels	
Single level	15 (33.3%)
Two levels	24 (53.4%)
Three levels	6 (13.3%)
Operative time (minutes)	132.7 ± 32.2
Preoperative lordosis (°)	8.7 ± 2.2

Values are presented as number, number (%), or mean ± standard deviation.

### Clinical outcomes

All patients reported at least partial improvement in pain scale and functional status during the last follow-up evaluation. NRS score improved from 6.3 (±0.4) to 2.1 (±0.1) and NDI score improved from 25.2 (±8.2) to 17.3 (±9.9). All scores (e.g., NRS and NDI) exhibited statistically significant improvement at the last follow-up postoperatively (*P* < 0.05). There were no statistically significant differences between the preoperative and last follow-up postoperative data in the modified AAOS-Modems disability outcome, PF, EF, EW, SF, BP, and GH (all *P *> 0.05) using the nonparametric Mann–Whitney *U* test (Table 2).

**Table 2:** Clinical outcomes of patients preoperatively and at last postoperative follow-up.

	Pre-op	Post-op	*P* value
NRS	6.3 ± 0.4	2.1 ± 0.1	0.000**
NDI	25.2 ± 8.2	17.3 ± 9.9	0.002*
Modified AAOS-Modems disability outcome tool spine-service version			
Vigorous activities	1.8 ± 1.5	1.8 ± 1.3	1.000
Moderate activities	1.9 ± 1.3	2.4 ± 1.5	0.387
Lifting or carrying groceries	2.2 ± 1.3	2.2 ± 1.3	1.000
Climbing several flights of stairs	3.0 ± 1.6	2.7 ± 1.8	0.613
Climbing one flight of stairs	3.6 ± 1.5	3.2 ± 1.9	0.190
Bending, kneeling, stooping	2.5 ± 1.5	2.7 ± 1.8	0.776
Walking more than 1.5 km	2.7 ± 1.8	3.3 ± 1.8	0.165
Walking several blocks	2.8 ± 1.7	3.3 ± 1.8	0.387
Walking one block	3.5 ± 1.5	3.9 ± 1.6	0.337
Sitting	3.2 ± 1.0	3.6 ± 1.3	0.273
Standing erect	2.8 ± 1.3	3.3 ± 1.4	0.273
Lying on back	2.8 ± 1.3	3.2 ± 1.0	0.436
Lying on stomach	3.0 ± 1.4	2.8 ± 1.5	0.721
Lying on sides	2.7 ± 1.4	2.8 ± 1.3	0.776
Grooming or bathing self	3.6 ± 1.5	3.3 ± 1.6	0.584
Sexual activities	2.2 ± 1.7	2.7 ± 1.8	0.273
Initiating gait	3.5 ± 1.7	3.8 ± 1.5	0.502
Crossing streetlights	3.8 ± 1.7	3.8 ± 1.7	1.000
SF-36			
Physical functioning	40.8 ± 20.8	44.1 ± 35.2	0.635
Energy fatigue	37.5 ± 22.8	37.5 ± 20.6	0.953
Emotional well being	51.7 ± 29.9	55.6 ± 27.5	0.944
Social functioning	52.5 ± 27.5	56.3 ± 34	0.610
Pain	33.0 ± 17.8	40.8 ± 27.4	0.326
General health	50.8 ± 22.7	40.5 ± 18.6	0.108

All data are presented as mean ± standard deviation (SD).

Pre-op, preoperative; Post-op, postoperative; NDI, Neck Disability Index; NRS, Numeric Rating Scale; SF-36, Short Form-36.

Significant difference: *P < 0.01, **P < 0.001 (paired t-test).

### Radiological outcomes

The fusion rate of patients undergoing ACDF following TSC was documented in 88.9% (40/45) of patients, and 93.3% (14/15) of patients achieved postoperative fusion in the one-level disease group, 87.5% (21/24) of patients with the two-level group, and 83.3% (5/6) of patients with the three-level group. There was no statistically significant difference in NRS and NDI scores between the fusion and no-fusion groups (Table 3).

**Table 3:** Clinical and radiological outcomes.

	Number (%)	*P* value
Fusion rate	40 (88.9%)	1.000
Single level	14 (93.3%)	
Two levels	21 (87.5%)	
Three levels	5 (83.3%)	
Fusion NRS	2.1 ± 1.1	1.000
No-fusion NRS	2.2 ± 0.4	
Fusion NDI	17.0 ± 9.7	0.490
No-fusion NDI	21.2 ± 8.3	
Subsidence	40 (88.9%)	1.000
Single level	14 (93.3%)	
Two levels	21 (87.5%)	
Three levels	5 (83.3%)	
Subsidence NRS	2.1 ± 1.1	0.381
No-subsidence NRS	1.4 ± 1.5	
Subsidence NDI	17.4 ± 9.5	0.942
No-subsidence NDI	17.6 ± 10.5	

NRS, Numeric Rating Scale; NDI, Neck Disability Index.

Cage subsidence was found in five patients (11.1%) at the last follow-up. No significant difference was found between single- and multilevel procedures in the incidence of cage subsidence. There was no statistically significant difference in NRS and NDI scores between the subsidence and no-subsidence groups. The cervical lordosis at the preoperative and last follow-up period was 8.7 ± 2.2° and 8.3 ± 3.2°, respectively (Table 3).

### Surgical complications

Seven patients had complications following TSC surgery, including dysphagia in one patient, nausea in two patients, sacrum pressure injury in one patient, wound issue in one patient, and chest pain in one patient. None of them underwent revision surgery.

## Discussion

We have demonstrated that in a cohort of patients undergoing TSC-based single- to multilevel fusion a strong basis for feasibility, safety, and preliminary efficacy for a device being currently used with APC fusion. Whilst no superiority claims are made over APC, we believe that our study forms a good basis for delivering Value-based care with potential for lower complications and potential improved cost-benefit.

APC as the standard technique in ACDF is effective in maintaining cervical stabilization, improving cervical lordotic alignment, preventing cage dislocation, and increasing fusion rates. Previous studies showed the efficacy and safety of using ACDF with cage and plate for signal level or multilevel patients with cervical spondylotic conditions (15, 17). However, increased complication rates associated with plate fixation have been reported in patients with multilevel ACDF (15, 17). In order to overcome these complications, stand-alone cages were developed and used. However, the understanding of these potential disadvantages (e.g., changes in cervical alignment, cage migration, low fusion rates, and the occurrence of subsidence) of using stand-alone cages for treating cervical spondylotic conditions remains incomplete (18). Compared to ACDF (e.g., APC), TSC could theoretically reduce the surgical trauma to soft tissues and reduce blood loss during the surgery, in single- and multilevel procedures. Our study achieved a good clinical efficacy (e.g., significant improvement in NRS and NDI scores) with TSC for single- and multilevel cervical spondylotic conditions.

Plate dislodgement, tracheoesophageal lesions, and dysphagia are recognized as the most occurred complications after ACDF using an additional anterior plate. Previous studies reported that the incidence of transient and chronic dysphagia following ACDF surgery ranges from 2% to 71% and from 3% to 21%, respectively (19). Transient dysphagia occurred in one patient that lasted 4–7 days in the present study (2.2%). None of the patients exhibited permanent dysphagia. A possible explanation for the occurrence of dysphagia following an anterior plate with APC is that the design and fixation of the anterior plate may lead to esophageal injury, soft tissue edema, hematoma, and adhesive formations around the plate. Reducing the use of implants is very important, which could avoid mechanical stimulus to the esophagus; furthermore, using a simple operative procedure and reducing the retraction of the esophagus can minimize the occurrence of postoperative dysphagia. Based on the minimally invasive procedure of TSC and the outcome of our results, we recommend the use of TSC for treatment of patients with cervical spondylotic conditions.

One advantage of plate fixation is early mobilization (20). TSC as an external soft collar is used for 3 weeks (one level), 6 weeks (two levels), and 8 weeks (three levels) (21). Our experience indicates that this does not cause the patients any undue discomfort. In fact, they feel psychologically reassured that their necks are being “taken care” of during the postoperative phase. The subaxial cervical spine moves through a lower arc of movement when compared to C0–C2 levels and further degenerative pathology assures global stiffness of the segments being treated; we feel this is sufficient for the early phase of healing. Prospective computational modeling to evaluate stability (that includes the role of neck muscles within collar immobilization) may further elucidate mechanics during TSC.

Fusion is the final aim of treating patients with cervical spondylotic conditions for ACDF or TSC. Previous studies reported similar rates of fusion between both APC and TSC in patients with cervical spondylotic conditions based on different involved levels (e.g., single-level cervical disease vs. multilevel cervical disease) (13), which is consistent with our results. Many issues have affected our results, such as the period of follow-up after surgical treatment, bone quality, different diagnoses of patients, preparation of the endplate for implanting the cage, and distraction achieved by the cage.

Subsidence is also considered the main complication of using the cage for fusion surgery, which has been reported in 9.3%–62.5% of patients with cervical spondylotic conditions (22). This study observed five patients (11.1%) with cage subsidence at the last follow-up. In theory, the subsidence of the cage may cause the disc height and foraminal height changes, which could cause the nerve root or spinal cord compression. The results of our study supported that TSC cannot significantly affect the NRS and NDI between the subsidence and no-subsidence groups. The authors recognize that subsidence is the outcome of numerous factors including bone quality and endplate preparation and may not be a consequence of cage-alone. Delayed union due to bone graft substitute may contribute to the occurrence of subsidence. However, in TSC, the one issue that is eliminated is stress protection afforded by plates and screws that may contribute to delayed union.

Sagittal misalignment as one of the main factors is important for balancing the stress distribution on internal fixation devices and maintaining cervical instability (22). We observed that TSC surgery can maintain cervical lordosis without a significant difference between single- and multilevel disease.

Several methodological issues require consideration. First, a small sample was included in the study. Second, the present study did not include a control group. Further multicenter randomized control trials in assessing TSC vs. APC techniques on the clinical efficacy and consequences of complications for treating patients with cervical spondylotic conditions are required.

## Conclusions

Stand-alone cage anterior cervical decompression and fusion surgery is an option for cervical degenerative disc disease of one, two, and three levels. This preliminary report can form the basis for a cost–benefit analysis study either prospectively or by way of meta-modeling comparing APC, LSC to TSC.

## Data Availability

The raw data supporting the conclusions of this article will be made available by the authors, without undue reservation.
